# Prevalence of obesity and its associated risk factors among the elderly in Malaysia: Findings from The National Health and Morbidity Survey (NHMS) 2015

**DOI:** 10.1371/journal.pone.0238566

**Published:** 2020-09-11

**Authors:** Suthahar Ariaratnam, Wan Shakira Rodzlan Hasani, Ambigga Devi Krishnapillai, Hamizatul Akmal Abd Hamid, Miaw Yn Jane Ling, Bee Kiau Ho, Sazlina Shariff Ghazali, Noorlaili Mohd Tohit, Muhammad Fadhli Mohd Yusoff

**Affiliations:** 1 Department of Psychiatry, Faculty of Medicine, Universiti Teknologi MARA (UiTM), Selangor, Malaysia; 2 Institute for Public Health, National Institutes of Health, Ministry of Health Malaysia, Setia Alam, Selangor, Malaysia; 3 Department of Family Medicine, Faculty of Medicine and Health, National Defence University of Malaysia, Kuala Lumpur, Malaysia; 4 Bandar Botanic Health Center, Bandar Botanic, Klang, Selangor, Malaysia; 5 Department of Family Medicine, Faculty of Medicine and Health Sciences, Universiti Putra Malaysia, Serdang, Malaysia; 6 Department of Family Medicine, Faculty of Medicine, Universiti Kebangsaan Malaysia, Bangi, Malaysia; San Raffaele Roma Open University, ITALY

## Abstract

**Background:**

Obesity is a crucial public health problem and is rising globally. This study was conducted to determine the prevalence of obesity and its associated factors among the elderly in Malaysia.

**Methods:**

Data were obtained from the National Health and Morbidity Survey (NHMS) 2015. This was a cross sectional population-based study using a two stage stratified random sampling design. Elderly population aged 60 years and above was selected. Data were collected using pre-tested self-administered questionnaire in the form of sociodemographic profile, non-communicable diseases (NCD) comorbidities (status on hypertension, diabetes and hypercholesterolemia) and NCD risk factors (current smoker and physical activity). Obesity has been defined using the World Health Organization (WHO) Body Mass Index (BMI) guideline, 1998. A complex sampling design analysis was used for the descriptive statistics. The associated risk factors for obesity were identified using Multiple Logistic Regression analysis.

**Results:**

A total of 3794 respondents were included in this study. The prevalence of obesity among Malaysian elderly was 30.2%. The prevalence of obesity among the elderly was significantly higher in females, respondents from urban areas and Indians. Approximately one third of the elderly with non-communicable diseases such as hypertension (33.1%) and diabetes (38.8%), respectively, were obese. Among elderly who were obese, majority of them (89.8%) had at least one NCD. The prevalence of obesity was 16.8% among current smokers (aOR 0.59). Multiple logistic regression analysis revealed that elderly females (aOR [adjusted odds ratio] 1.52), having secondary education (aOR 1.96) with household income of RM 3000 and above (aOR 1.57) as well as being hypertensive (aOR 1.61) and diabetic (aOR 1.50) were more likely to be obese. In contrast, the Chinese elderly respondents (aOR 0.62) and current smokers (aOR 0.59) were less likely to be obese. There were no significant associations of obesity with hypercholesterolemia or with physical activity.

**Conclusions:**

A substantial proportion of Malaysian elderly were obese, and factors associated with obesity among them were being female, having secondary education with a household income of RM 3000 and above and being hypertensive or diabetic. Enhanced health promotion and education should be targeted at younger people in order to prevent obesity in the later years.

## Introduction

Obesity poses a major public health problem and its prevalence is rising globally [1]. The World Health Organization (WHO) defines overweight and obesity as persons having Body Mass Index (BMI) equal to or greater than 25 and 30, respectively. The prevalence of obesity worldwide has doubled from 6.4% in 1980 to 12.0% in 2008 [2]. Overweight and obesity were estimated to be responsible for 3.4 million deaths and 3.9% of years of life lost globally [3]. Moreover, overweight and obesity persons were also found to be associated with various non-communicable diseases (NCD), such as hypertension, type 2 diabetes, coronary heart disease, stroke, gallbladder disease, osteoarthritis, sleep apnea and respiratory problems [2].

Although obesity was a health problem across all age groups, the number of obese older adults had increased significantly [4]. Obesity among the elderly is of great concern as it can be associated with functional limitations due to decreased muscle mass and strength, increased joint dysfunction, disabilities in activities of daily living, frailty, chronic pain and impaired quality of life [4]. Furthermore, obesity in the middle age has been associated with a higher chance of developing dementia among the older age group in the United States [5]. However, despite the association of obesity with various types of morbidity, there are still ongoing debates of whether obesity is harmful to the elderly [6].

A descriptive cross sectional survey conducted among only fifty three elderly in a small town in Selangor, Malaysia found that the prevalence of obesity was 30% [7] while another Malaysian study conducted between 2007 to 2008, reported the prevalence as 19.3% [8].

To look for functional limitations associated with obesity in Malaysia, we undertook a nationwide survey to explore the factors associated with obesity among elderly individuals using the data from the National Health and Morbidity Survey (NHMS), 2015 conducted in Malaysia [9].

## Methods

This study was conducted using secondary data from the National Health and Morbidity Survey (NHMS) that was conducted in 2015 [9]. The NHMS was a cross sectional population-based study of Malaysian population who were non-institutionalized and residing in Malaysia for at least 2 weeks prior to the data collection. Two stage stratified random sampling was applied. The sampling was stratified by states and location (urban and rural area). The Primary Sampling Unit (PSU) was the Enumeration Blocks (EB) and the Second Sampling Unit (SSU) was Living Quarters (LQs) within the selected EBs. All household members within the selected LQs were included in the study. Institutional population such as those staying in hotel, hostel or hospital were excluded from the survey. For this study, data for elderly population (60 years old and above) were selected.

The sample size for NHMS was calculated using a single proportion formula for estimation of prevalence, the optimum sample size required was 10,428 living quarters. The pre-tested and piloted self-administered questionnaire was used for the data collection (refer to S1 Appendix).

The dependent variable was the obesity status. The respondent’s weight was measured using the digital weighing machines (TANITA HD-319) and the height was measured in centimetre using SECA 206 Bodymeter. BMI was then calculated and categorized using WHO BMI guideline, 1998: (<25 kg/m2 as underweight to normal weight, 25.0 to 29.9 kg/m2 as overweight and ≥30 kg/m2 as obesity). The associated factors (independent variables) were socio-demographic characteristic (location, gender, ethnicity, education level and household income), NCD comorbidities (status on hypertension, diabetes and hypercholesterolemia) and NCD risk factor (current smoker and physical activity).

The NCD comorbidities status was obtained using the screening questionnaire and clinical measurement. The Omron Digital Automatic Blood Pressure Monitor Model HEM-907 was used for measurement of blood pressure. Blood pressure was recorded as an average reading from two electronic pressure monitoring measurements. Respondents were classified as having hypertension if their blood pressure was ≥140mmHg systolic or ≥90mmHg diastolic or they were told to have hypertension by medical personnel previously. The validated finger-pricked (from capillary blood) CardioCheck portable blood test system was used to measure total cholesterol and blood glucose level. Using the WHO, 1999 definition of Diabetes Mellitus, respondents were classified accordingly as having the condition if they have been told to have diabetes by medical personnel or when the respondents were not known to have diabetes but had ≥FBS 7.0 mmol/L or RBS≥ 11.1 mmol/L. Similarly, the respondents were classified as having hypercholesterolemia if they were known hypercholesterolemia from the screening question and those who had total blood cholesterol of 6.2 mmol/L or more during the survey [10].

The smoking status was defined as current smoker when the response was “Yes” to smoking any tobacco product daily or occasionally. For the physical activity, the seven-day history of physical activity was asked of the respondent using short International Physical Activity Questionnaire (IPAQ) [11]. Metabolic Equivalent of Task (MET) from the diary of 7 days were calculated and categorized as physical active and not active. The following criteria were considered as being physically active: 1) ≥3 days of vigorous activities for at least 20 minutes per day Or 2) ≥5 days of moderate intensity activities and walking of at least 30 minutes per day Or 3) ≥5 days of any combination of walking, moderate intensity and vigorous intensity activities achieving a minimum of at least 600 MET-minute /week Or 4) Vigorous intensity activities for at least 3 days and accumulating at least 1500 MET-minute/week Or 5) ≥7 days of any combination of walking, moderate intensity and vigorous intensity activities achieving a minimum of at least 3000 MET-minute/week. Those who did not meet the above criteria were considered as being physically inactive.

Those selected variables were extracted from NHMS’s data and was analysed using Statistical Package for the Social Sciences (SPSS) version 21. A complex sampling design analysis was used for descriptive statistics. The associated factor for obesity was identified using Multiple Logistic Regression analysis. On the basis of univariable analysis (crude odd ratio from simple logistic regression), the variables with p<0.25 and considered for biological plausibility were included in the multivariable model (multiple logistic regression analysis). The Multivariable model was obtained based on a backward likelihood ratio method. Multicollinearity problem and all possible two-way interaction terms were checked one by one together with the main effect model. Model fitness using goodness of fit statistics was used to assess the fit of logistic model against actual outcomes. This study had obtained the ethical approval from the National Medical Research Registry (NMRR), Ministry of Health Malaysia (Registration no. NMRR-14-1064-21877).

## Results

A total of 3794 of elderly were included in this study. More than half of the respondents were female (53.3%) and hailed from the rural area (51.2%). By ethnicity, 64.0% were Malays, 21.9% were Chinese, 6.8% were other Bumiputeras, 6.1% were Indians while 1.5% were categorized as other ethnicity. Half of the respondents had at least primary education and only 20.3% had no formal education. Majority of the respondents had a household income of less than RM 1000.

Prevalence of obesity among the Malaysian elderly was 30.2% (95% CI: 27.9, 32.6). By socio-demographic profiling, the prevalence of obesity among the elderly was significantly lower in males compared to females. Prevalence of obesity was significantly higher among urban dwellers (32.1%; 95% CI: 29.1, 35.3). The highest prevalence of obesity was among the Indians (52.6%; 95% CI: 44.4, 60.7), followed by the Malays (33.3%; 95% CI: 30.6, 36.2) and Chinese (24.6%, 95% CI: 20.6, 29.1). The prevalence of obesity among current smokers was 16.8% (95% CI: 13.2, 21.3). Approximately one third with hypertension (33.1%), diabetes (38.8%) and hypercholesterolemia (32.4%), respectively, were obese. Among elderly who were obese, majority of them (89.8%) had at least one NCD. Detailed prevalence rates are shown in Table 1.

**Table 1:** Prevalence of obese and non-obese participants based on socio-demographic and non-communicable diseases profiles.

Sociodemographic Characteristics	Obese					Non Obese				
	Prevalence (%)	Count	95% CI		Estimated Population	Prevalence (%)	Count	95% CI		Estimated Population
			Lower	Upper				Lower	Upper	
***Malaysia***	30.2	1040	27.9	32.6	707905	69.8	2366	67.4	72.1	1636258
***Location***										
Urban	32.1	533	29.1	35.3	541253	67.9	1122	64.7	70.9	1143509
Rural	25.3	507	22.8	28.0	166651	74.7	1244	72.0	77.2	492749
***Sex***										
Male	26.4	409	23.5	29.5	306089	73.6	1195	70.5	76.5	853235
Female	33.9	631	30.9	37.1	401816	66.1	1171	62.9	69.1	783022
***Ethnicity***										
Malays	33.3	716	30.6	36.2	372432	66.7	1458	63.8	69.4	746053
Chinese	24.6	180	20.6	29.1	203043	75.4	556	70.9	79.4	622621
Indians	52.6	81	44.4	60.7	83776	47.4	129	39.3	55.6	75419
Other Bumiputras	21.0	51	15.2	28.4	44234	79.0	184	71.6	84.8	165910
Others	14.4	12	5.4	33.3	4420	85.6	39	66.7	94.6	26255
***Education Level***										
No formal education	20.9	144	16.7	25.8	95199	79.1	509	74.2	83.3	360291
Primary education	31.6	562	28.8	34.6	354457	68.4	1219	65.4	71.2	766963
Secondary education	33.9	265	29.6	38.5	197956	66.1	500	61.5	70.4	386258
Tertiary education	32.7	61	24.9	41.6	52920	67.3	118	58.4	75.1	108877
***Income Group***										
Less than RM 1000	26.4	328	23.0	30.1	210558	73.6	890	69.9	77.0	587416
RM 1000—RM 1999	28.6	214	24.2	33.5	131847	71.4	523	66.5	75.8	328590
RM 2000—RM 2999	26.8	139	21.6	32.6	79772	73.2	329	67.4	78.4	218207
RM 3000 & above	36.3	359	32.4	40.4	285727	63.7	624	59.6	67.7	502044
***Hypertension***										
Yes	33.1	809	30.4	35.9	545105	66.9	1575	64.1	69.6	1101535
No	23.3	231	19.9	27.1	162800	76.7	791	72.9	80.1	534722
***Diabetes***										
Yes	38.8	436	34.8	42.9	305046	61.2	703	57.1	65.2	481760
No	25.9	604	23.3	28.7	402858	74.1	1663	71.3	76.7	1154498
***Hypercholesterolemia***										
Yes	32.4	537	29.3	35.6	335157	67.6	1056	64.4	70.7	699429
No	28.5	503	25.6	31.5	372748	71.5	1310	68.5	74.4	936824
***Physical activity***										
Active	32.6	611	29.6	35.7	409936	67.4	1280	64.3	70.4	848978
Inactive	27.8	425	24.6	31.2	295630	72.2	1057	68.8	75.4	766920
***Current Smoker***										
Yes	16.8	94	13.2	21.3	50618	83.2	415	78.7	86.8	250006
No	32.2	945	29.6	34.8	656603	67.8	1949	65.2	70.4	1385070

Factors associated with obesity among the elderly are presented in Table 2. Multiple logistic regression analysis revealed that the elderly females were 1.52 times (95% CI: 1.28, 1.81) more likely to be obese as compared to elderly males. By ethnicity, Chinese elderly were less likely to be obese (aOR: 0.62; 95% CI: 0.50, 0.75) as compared to Malay elderly. Elderly who had primary education (aOR: 1.74; 95% CI: 1.39, 2.18), secondary education (aOR 1.96; 95% CI: 1.51, 2.54) and tertiary education (aOR 1.83; 95% CI: 1.23, 2.70) attainment had higher odds of being obese compared to those with no formal education. By income group, elderly who earned RM 3000 and above (aOR 1.57; 95% CI: 1.29, 1.91) were more likely to be obese compared to those who earned less than RM 1000. Elderly who had hypertension (aOR 1.61; 95% CI: 1.35, 1.93) and diabetes (aOR 1.50; 95% CI: 1.28, 1.76) were at higher risk of being obese than those who had no such diseases. As for smoking status, elderly current smokers were 41% (aOR 0.59, 95% CI: 0.45, 0.77) less likely to be obese compared to non-smokers. Pertaining to physical activity, elderly who were physically inactive were 22% less likely to be obese (aOR 0.78, 95% CI: 0.67, 0.91). There were no significant association of obesity with hypercholesterolemia.

**Table 2:** Factor associated with obesity among elderly (60 years old and above) using logistic regression (n = 3794).

	*Simple Logistic Regression (SLR)*			*Multiple Logistic regression (MLR)* *		
	*b*	Crude OR (95% CI)	P-value	*b*	Adjusted OR (95% CI)	P-value
*Location*						
Urban	1					
Rural	-0.15	0.86 (0.74,0.99)	0.040	-0.11	0.89 (0.76, 1.06)	0.193
*Sex*						
Male	1			1		
Female	0.45	1.58 (1.36,1.83)	<0.001	0.42	1.52 (1.28,1.81)	<0.001
*Ethnicity*						
Malays				1		
Chinese	-0.42	0.66 (0.55,0.80)	<0.001	-0.49	0.62 (0.50, 0.75)	<0.001
Indians	0.25	1.28 (0.96, 1.71)	0.099	0.05	1.10 (0.81, 1.50)	0.543
Other Bumiputras	-0.57	0.56 (0.41,0.78)	0.001	-0.31	0.73 (0.52, 1.03)	0.077
Others	-0.47	0.63 (0.33, 1.21)	0.161	-0.42	0.66 (0.32, 1.36)	0.254
*Education Level*						
No formal education	1			1		
Primary education	0.49	1.63 (1.32,2.01)	<0.001	0.55	1.74 (1.39, 2.18)	<0.001
Secondary education	0.63	1.87 (1.48,2.38)	<0.001	0.67	1.96 (1.51, 2.54)	<0.001
Tertiary education	0.60	1.83 (1.28, 2.62)	0.001	0.60	1.83 (1.23, 2.70)	0.003
*Income Group*						
Less than RM 1000	1			1		
RM 1000—RM 1999	0.11	1.11 (0.91,1.36)	0.313	0.04	1.05 (0.85, 1.30)	0.686
RM 2000—RM 2999	0.14	1.15 (0.91, 1.45)	0.255	0.07	1.07 (0.83, 1.36)	0.607
RM 3000 & above	0.45	1.56 (1.30,1.87)	<0.001	0.45	1.57 (1.29,1.91)	<0.001
*Hypertension*						
No	1			1		
Yes	0.57	1.76 (1.49, 2.08)	<0.001	0.48	1.61 (1.35, 1.93)	<0.001
*Diabetes*						
No	1			1		
Yes	0.54	1.71 (1.47, 1.99)	<0.001	0.40	1.50 (1.28, 1.76)	<0.001
*Hypercholesterolemia*						
No	1					
Yes	0.28	1.32 (1.14, 1.53)	<0.001	0.08	1.08 (0.93, 1.27)	0.317
Physical activity						
Active	1			1		
Inactive	-0.17	0.84 (0.73, 0.98)	0.023	-0.25	0.78 (0.67,0.91)	0.002
*Current Smoker*						
No	1			1		
Yes	-0.76	0.47 (0.37,0.59)	<0.001	-0.53	0.59 (0.45, 0.77)	<0.001

*Multiple Logistic regression was applied. Final model was adjusted for sex, ethnicity, education level, income group, hypertension, diabetes, physical activity, current smoker. Multicollinearity and interaction were checked and not found. Hosmer Lemeshow test P value = 0.984, Classification Table (overall correctly classified percentage = 69.7%) and ROC curve (area under ROC curve = 65.7%) were accepted to check model fitness.

## Discussion

To the best of our knowledge this is the first study evaluating obesity and its associated factors among the elderly in Malaysia using a national representative sample.

Pertaining to gender, the prevalence of obesity in the elderly was significantly lower among male than female subjects. This result conformed with other reports [12–15] which had found that females had more predilection for obesity compared to males.

Ethnicity profiling showed that Chinese elderly were less likely to be obese compared to Malay elderly. This could be ascribed to dietary habits among them. Malays who are Muslims tend to choose food which are permissible (*halal* or lawful). Thus, influencing their food consumption [16]. There was no significant association of obesity for the Indians when compared to the Malay elderly individuals.

In terms of education status, this study demonstrated that the elderly who had primary education, secondary education and tertiary education attainments had higher odds of being obese compared to those with no formal education. There had been conflicting results pertaining to this finding as one study [12] reported no significant association between elderly obesity and education level while Sabanayagam et al. [17] found that low education level was a risk factor for obesity among the Malays in Singapore. This discrepancy may be due to the variance in sample size, medical comorbidities, dietary habits, socioeconomic status and other confounders of obesity.

Regarding income differences, elderly individuals who earned RM 3000 and above were more likely to be obese compared to those who earned less than RM 1000. This finding was in line with other studies focussing on the elderly population [12, 18] which had affirmed that higher income earners among the elderly were more at risk of being obese as they tended to lead a sedentary life style, more inclined to eat food rich in fat and indulge in less exercise, all of which may lead to increase weight and hence obesity.

By NCD category, elderly who had hypertension and diabetes were at higher risk of being obese than those without such diseases. This outcome was in agreement with numerous studies that concluded NCD was indeed a significant risk factor for developing obesity in the elderly [19, 20].

Our study further discovered that physical activity was not significantly negatively associated with obesity. We speculate that our elderly population did not adhere meticulously to their dietary consumption in terms of specific food intake though their physical activity was deemed to be active.

The strength of our study lies in its large sample size though confined to the elderly population. We acknowledge several limitations in this study. First, psychosocial factors such as life events and level of social support can have an impact on the outcome of our study, but these were not investigated in this study. Second, this being a cross sectional study did not allow for cause and effect relationships to be studied. Third, the scope of this study did not include dietary intake among respondents. The per-capita sugar consumption in Malaysia was almost 40kg/year [21] and this could have regrettably contributed to the obesity prevalence.

## Conclusions

This study highlights an alarming situation of the prevalence of obesity among Malaysian elderly. Female elderly subjects with NCD risk factors such as hypertension and diabetes were more likely to be obese. Enhanced health promotion and education should be targeted at younger age groups, as preventive measures to combat obesity later in life.

In addition, workable and comprehensive intervention to curb obesity should start immediately among the elderly population. Suitable activities and weight loss intervention programs should be incorporated into community settings as reduction in weight had significantly lowered the risk for NCD comorbidities.

## Supporting information

S1 Appendix (DOCX)
