# Supplementary material for: Prevalence of obesity and its associated risk factors among the elderly in Malaysia: Findings from The National Health and Morbidity Survey (NHMS) 2015
Source: PLoS One. 2020 Sep 11;15(9):e0238566. doi: 10.1371/journal.pone.0238566 (PMC7486079; doi:10.1371/journal.pone.0238566)
Supplement: S1 Appendix — (DOCX) [file pone.0238566.s001.docx]

**S1 Appendix**
